# Navigation Aiding by a Hybrid Laser-Camera Motion Estimator for Micro Aerial Vehicles

**DOI:** 10.3390/s16091516

**Published:** 2016-09-16

**Authors:** Jamal Atman, Manuel Popp, Jan Ruppelt, Gert F. Trommer

**Affiliations:** 1 Institute of Systems Optimization (ITE), Karlsruhe Institute of Technology (KIT), Karlsruhe 76049, Germany; manuel.popp@kit.edu (M.P.); jan.ruppelt@kit.edu (J.R.); gert.trommer@kit.edu (G.F.T.); 2 ITMO University, Saint Petersburg 197046, Russia

**Keywords:** hybrid sensor, laser-camera calibration, ego-motion estimation, covariance estimation, integrated navigation system

## Abstract

Micro Air Vehicles (MAVs) equipped with various sensors are able to carry out autonomous flights. However, the self-localization of autonomous agents is mostly dependent on Global Navigation Satellite Systems (GNSS). In order to provide an accurate navigation solution in absence of GNSS signals, this article presents a hybrid sensor. The hybrid sensor is a deep integration of a monocular camera and a 2D laser rangefinder so that the motion of the MAV is estimated. This realization is expected to be more flexible in terms of environments compared to laser-scan-matching approaches. The estimated ego-motion is then integrated in the MAV’s navigation system. However, first, the knowledge about the pose between both sensors is obtained by proposing an improved calibration method. For both calibration and ego-motion estimation, 3D-to-2D correspondences are used and the Perspective-3-Point (P3P) problem is solved. Moreover, the covariance estimation of the relative motion is presented. The experiments show very accurate calibration and navigation results.

## 1. Introduction

At present time, Micro Air Vehicles (MAVs) are becoming very popular in various applications. They are affordable and flexible in terms of location due to their size and weight. Equipping them with additional sensors, MAVs are able to carry out autonomous flights. Thus, MAVs are able to explore areas autonomously that are difficult to access by pedestrians or ground vehicles. This can be for instance disaster areas, where the MAV provides rescue teams with mission-specific information. In order to execute such missions, a very accurate navigation solution at all times is required. Typically, MAVs are equipped with Global Navigation Satellite System (GNSS) receivers, which provide absolute position and aid the Inertial Navigation System (INS). However, this kind of navigation system is not sufficient because the MAV should be able to operate also in GNSS-denied areas, such as indoor environments. Moreover, urban canyons, multipath propagation and jamming are reasons for low GNSS positioning accuracy. Therefore, other sensors, like a camera and a laser rangefinder, are required. Those sensors do not provide absolute position information, but they increase the robustness and accuracy of the navigation system. The camera detects features easily, which are tracked so that ego-motion can be observed. Nevertheless, the scale factor with respect to metric dimensions is unknown due to the absence of depth information. The extensively-used visual odometry technique provides approaches to solve this problem. For example, either an Inertial Measurement Unit (IMU) [1] or stereo camera [2] can be used. Both methods are limited. The IMU filter approach needs variation in camera perspective in order to decrease the uncertainty of 3D points. The depth accuracy of the stereo camera approach is limited by its baseline [3]. By contrast, laser rangefinders provide accurate depth information without triangulation. However, the drawback is that corresponding features between subsequent laser scans are in general hardly detectable. In addition, it is even more difficult as the MAV has six degrees of freedom. There are several laser-scan-matching approaches, but they mostly expect structured environments, such as indoor scenes (e.g., [4]). In order to become more flexible in terms of the MAV’s surroundings, this article deals with fusing both complementary sensors at the early stage so that their advantages are combined. This requires good knowledge about the pose between both sensors. Thus, an extrinsic calibration is needed.

The literature on laser-camera calibration shows a variety of approaches. They differ in the kind of correspondences established between both sensors. An overview is given in [5]. First, many publications are based on the approach of Zhang and Pless [6], where plane-point correspondences are found. The idea is that the plane of a checkerboard is described in camera coordinates. Knowing that the plane contains also the laser points, the constraints per pose are obtained. At least five poses are needed to calibrate the laser-camera system. Second, line-point correspondences are used, for instance in [7], where the calibration object has a triangular shape. The constraint is that the laser points on the edges correspond to extracted edges in the image. Third, in [8], the calibration method of Zhang and Pless [6] is enhanced. The approach finds plane-line correspondences in dual space. Besides the aforementioned kind of correspondences, Hoang et al. [9] presents a calibration object that establishes point-point correspondences. Due to the geometry of the calibration object, 3D feature points of the laser rangefinder are recovered in image coordinates. The pose between both sensors is obtained by solving the Perspective-3-Point (P3P) problem [10]. According to Li et al. [7], who compared different feature points, point features lead to the most accurate results. Because of this finding, the approach of Hoang et al. was chosen. Moreover, the P3P problem describes the minimal problem, where a finite number of solutions exist. This is advantageous when implementing a robust model estimation optimally by using the smallest possible subset. The chosen approach is further improved in order to obtain very accurate calibration results.

After knowing the pose between both sensors, the relative motion of the MAV is estimated. The authors of [11] propose an approach that estimates the depth information of image features, which do not necessarily lie on the projected laser line. This is possible by assuming ground vehicle motion and a structured environment, such as indoors. In [12], the pixels with depth information are used in addition to the image features. The features are tracked by optical flow, and the relative motion is obtained by bundle adjustment. On the other hand, the authors of [13] solve the P3P problem in order to gain relative motion. In this work, they equipped a ground vehicle with a vertical laser in the lateral direction and a camera system of six cameras. The estimated relative motion between adjacent frames is only accumulated and not integrated in a navigation system. The positioning is then refined by using manually-detected loop closures. This is not suitable for online navigation processing. In one of our previous papers, pixels with depth information are also used [14]. However, this is focused on SLAM (Simultaneous Localization and Mapping) and the total navigation system of the MAV. To the authors’ best knowledge, in previous studies, deeply-integrated laser-camera systems have been scarcely investigated from the point of view of integrated navigation systems.

In this article, a hybrid laser-camera sensor for navigation aiding is presented. The objective is to obtain an accurate navigation solution and become more flexible in terms of the MAV’s surroundings compared to the existing laser-scan-matching approach [4]. This includes many aspects from the deep integration of two complementary sensors, to ego-motion estimation, to the final integrated navigation system. Both deep integration, namely the laser-camera calibration and ego-motion estimation use 3D-to-2D correspondences. Hence, applying the algorithm proposed by Kneip et al. [15] in a RANSAC (Random Sample and Consensus [10]) scheme, the P3P problem is solved. The solution is further refined by nonlinear optimization. The calibration results obtained by simulation and experiments show that a precise knowledge about the pose between both sensors is gathered. Similarly, the ego-motion estimation is very accurate. Moreover, this article deals with the covariance estimation of the estimated relative motion. It is shown that the estimated uncertainty correlates with the actual errors. This results in a more accurate and robust navigation solution.

## 2. System Overview

**Figure 1:**
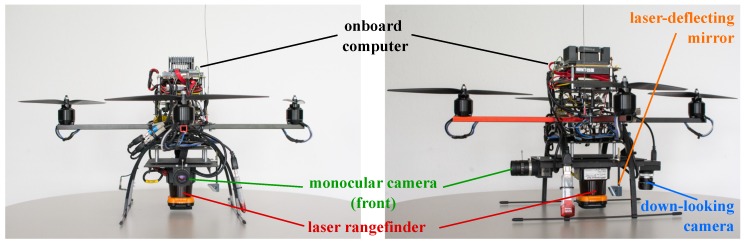
The Micro Air Vehicle (MAV) equipped with various sensors.

The presented MAV was developed by the Institute of Systems Optimization at Karlsruhe Institute of Technology. Our MAV is equipped with various sensors. The experimental platform is shown in Figure 1. In this article, particular attention is paid to the 2D laser rangefinder manufactured by Hokuyo (UTM-30LX) and the front-facing camera (IDS UI-1240SE). The Inertial Measurement Unit (IMU) consists of a triaxial accelerometer by Analog Devices (ADIS 16255) and three gyroscopes (VTI SCA3100). The embedded computer (Adlink, Cool XpressRunner GS-45 Intel Core 2 Duo (2.26 GHz)) processes the developed algorithms.

## 3. Coordinate Systems and Transformations

**Figure 2:**
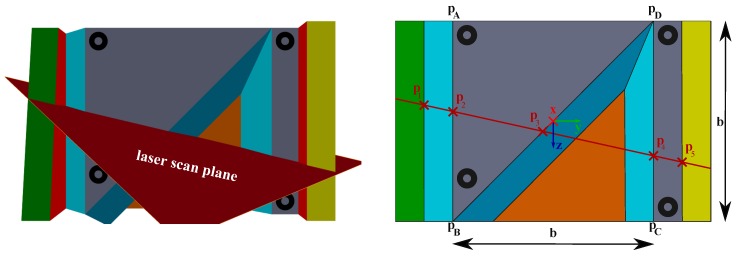
3D model sensed by the camera and laser rangefinder (**left**) and its schematic representation (**right**).

The coordinate systems used in this article are defined as follows:

- Body frame (${}^{b}$): The origin is located in the MAV’s center of mass. The *x*-axis is directed forwards; *y* is the right axis; and the *z*-axis points downwards.
- Navigation frame (${}^{n}$): The origin is coincident with the origin of body frame. The *x*-axis is directed to north; *y* is the east direction; and the *z*-axis is the vertical component.
- Laser frame (${}^{l}$): The *x*-axis is directed forwards; *y* is the right axis; and the *z*-axis points downwards; whereas the laser scan plane lies in z=0.
- Camera frame (${}^{c}$): The origin is located in the optical center. The *z*-axis is directed forwards; *x* is the right axis; and the *y*-axis points downwards.
- Calibration object frame (${}^{o}$): The center of the diagonal in the front plane of the calibration object defines the origin; see Figure 2 (right). The *x*-axis is directed forwards; *y* is the right axis; and the *z*-axis points downwards.

*x*, ***x*** and X represent a scalar value, a vector and a matrix, respectively. The matrix $C_{\alpha }^{\beta }$ defines the rotation between the *α*-coordinate system to the *β*-coordinate system. The translation between coordinate systems *α* and *β* in *γ*-coordinates is represented by $t_{\alpha \beta }^{\gamma }$.

## 4. Calibration

In order to use both complementary sensors, namely a monocular camera and a 2D laser rangefinder, as a hybrid sensor, it is essential to have accurate knowledge about the pose to each other. Due to this pose information, $C_{c}^{l}$ and $t_{lc}^{l}$, accurate laser rangefinder readings can be assigned to image pixels of the calibrated camera.

### 4.1. Calibration Object

First of all, the calibration object and a possible sensor configuration are presented in Figure 2. The calibration object consists of two main parallel planes (grey and orange), whereas the back plane (orange) is visible as a right-angled triangle. Similarly, Hoang et al. [9] proposed this kind of calibration object. Apart from that, two more modifications were realized. First, four circular rings were attached to the front plane. Second, ramps connecting the front (grey) and back plane were established. The motivation of those modifications will be described later on.

As mentioned before, Figure 2 shows a possible sensor configuration seen from the camera perspective. The camera detects the circular rings easily, and the laser scan plane intersects with the calibration object. Since depth discontinuities will occur, particularly points $p_{1}$ to $p_{5}$ can be extracted from the laser depth readings. A detailed description of feature extraction follows in the next sections.

As laser points are not in general visible to the camera, the coplanar circular rings make it possible to recover them in the image. Thus, 3D-to-2D correspondences can be obtained for the P3P-algorithm, which calculates the pose between both sensors.

To sum up, a successful calibration can be achieved, when the camera’s field of view includes all four circular rings and the laser scan plane includes at least the relevant points $p_{2}$ to $p_{4}$.

### 4.2. 3D Laser Feature Extraction

Because of the local depth discontinuity intersection, points $p_{2}$ to $p_{4}$ are suitable to choose as 3D laser feature points. Once extracted, those points can be directly described in laser coordinates.

This approach is also described by Hoang et al. [9]. However, the actual implementation is scarcely mentioned. For our calibration method, the Adaptive Line Extraction (ALE) [16] was chosen. In the presence of noise, this method shows robust performance regarding the problem that true line segments are falsely broken [17]. Thus, laser points on the calibration object can be described as line segments, and their endpoints include $p_{2}$ to $p_{4}$. Consequently, the relevant points are determined with all laser points that correspond to the individual line segments. On the contrary, depth gradient approaches would only depend on the points on the edges, where laser artifacts or ambiguous solutions can be expected.

However, an accurate extraction of points located on edges cannot be assured, since the horizontal discretization of laser range measurements is not negligible. Attaching the ramps to the calibration object, this problem can be solved. In order to get edge points, the intersection of line segments, found by the ALE algorithm, is calculated. For this purpose the line segments are recalculated by using only the inner points in order to obtain a better parametrization of lines.

**Figure 3:**
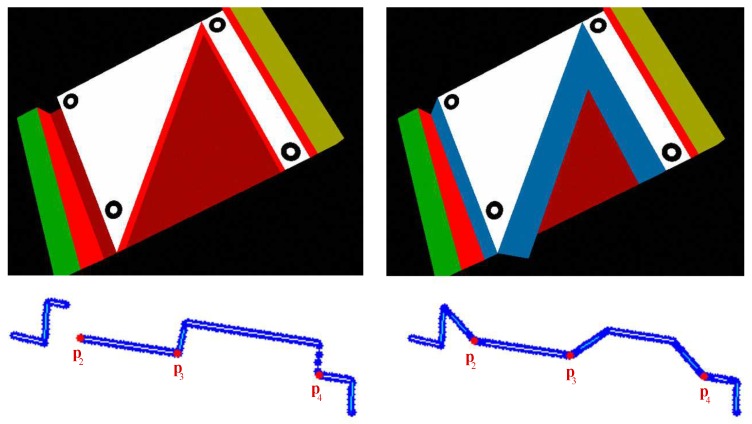
Comparison of laser depth readings between the original [9] (**left**) and our modified calibration object (**right**).

A comparison between two laser range readings caused by the original and the modified calibration object is depicted in Figure 3.

This indicates that due to the ramps, all relevant line segments are visible from various view points, so that an accurate point estimation can be achieved.

### 4.3. 2D Corresponding Camera Feature Point

After finding the relevant points $p_{2}^{l}$ to $p_{4}^{l}$ in laser coordinates, it is now necessary to recover the corresponding points, especially $p_{3}$, in the image. This is possible because of the geometry and the circular markers of the calibration object. First, the points are described in object coordinates by simple geometrical calculations. Second, the points will be found in image coordinates by using the circular rings (see Section 4.3.2).

#### 4.3.1. Geometric Calculation

Point $p_{3}$ can be simply calculated by the intercept theorem. The intersection point $p_{3}$ is caused by lines through $p_{B}$, $p_{D}$ and $p_{2}$, $p_{4}$, respectively. The distances $p_{23}$, $p_{34}$ are given by the extracted laser feature points, where the distances are defined by:

(1) $$p_{ij}:=∥p_{i}^{l}-p_{j}^{l}∥=∥p_{i}^{c}-p_{j}^{c}∥$$

**Figure 4:**
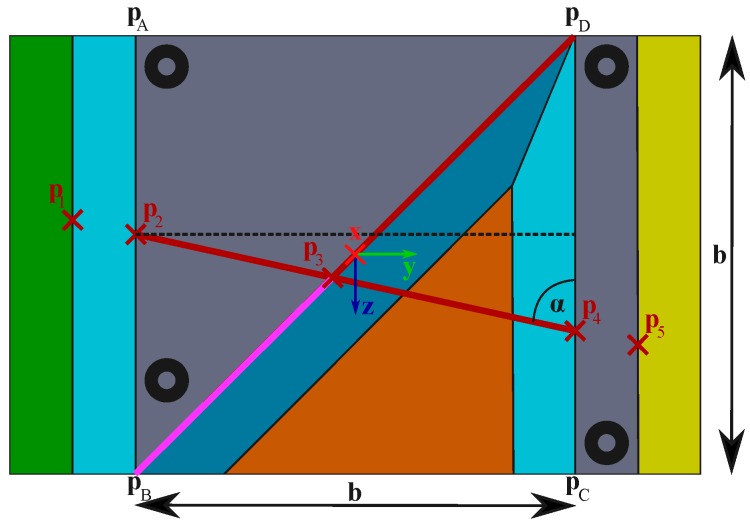
Relevant segments in order to determine points in calibration object coordinates.

In addition, the length of the diagonal is also known by $\sqrt{2}b$. Thus, the marked distance in Figure 4 can be calculated by:

(2) $$p_{B3}=\frac{p_{23}\;\cdot \;\sqrt{2}b}{p_{23}+p_{34}}$$

It is clear that from Equation (2), $p_{3}$ in object coordinates can be obtained by:

(3) p3o=p3,xop3,yop3,zo=0−pB32−b2−pB32+b2

Beyond that, Hoang et al. [9] suppose the calculation of $p_{2}$ and $p_{4}$ in object coordinates. Using the law of cosines, the distances:

(4) $$\begin{array}{cc} p_{B2} & =\sqrt{p_{B3}^{2}+p_{23}^{2}-2\;\cdot \;p_{B3}\;\cdot \;p_{23}\;\cdot \;\cos\left(\pi -\frac{\pi }{4}-\alpha \right)}\\ p_{D4} & =\sqrt{{\left(\sqrt{2}b-p_{B3}\right)}^{2}+p_{34}^{2}-2\;\cdot \;\left(\sqrt{2}b-p_{B3}\right)\;\cdot \;p_{34}\;\cdot \;\cos\left(\pi -\frac{\pi }{4}-\alpha \right)} \end{array}$$

can be calculated.

Since the determination of angle:

(5) $$\alpha =\left\{\begin{array}{c} \arcsin\left(\frac{b}{p_{24}}\right)\\ \pi -\arcsin\left(\frac{b}{p_{24}}\right) \end{array}\right.$$

illustrated in Figure 4, is ambiguous, two possible solutions for $p_{2}^{o}$ and $p_{4}^{o}$ respectively exist. As the P3P algorithm is embedded in a RANSAC scheme, outliers are detected, so that the incorrect hypothesis can also be used as the input. Eventually, the hypotheses from Equation (4) can be computed by:

(6) $$p_{2}^{o}=\left(\begin{array}{c} p_{2,x}^{o}\\ p_{2,y}^{o}\\ p_{2,z}^{o} \end{array}\right)=\left(\begin{array}{c} 0\\ -\frac{b}{2}\\ \frac{b}{2}-p_{B2} \end{array}\right)$$

and:

(7) $$p_{4}^{o}=\left(\begin{array}{c} p_{4,x}^{o}\\ p_{4,y}^{o}\\ p_{4,z}^{o} \end{array}\right)=\left(\begin{array}{c} 0\\ b-\frac{b}{2}\\ -\left(b-p_{D4}-\frac{b}{2}\right) \end{array}\right)$$

**Figure 5:**
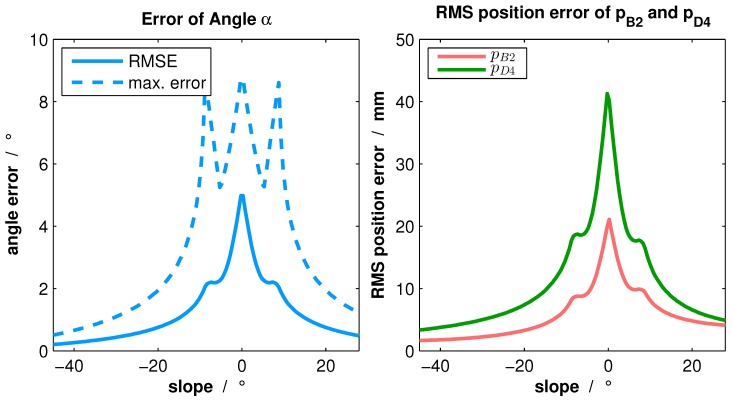
Angle estimation error due to small errors of 3D feature point extraction (**left**) and the resulting segment errors (**right**).

Nevertheless, only the 3D-to-2D correspondences of $p_{3}$ per pose are used for our calibration. Small errors in determining the 3D feature points could cause non-acceptable position errors of $p_{2}^{o}$ and $p_{4}^{o}$ in object coordinates. Concerning this issue, those randomly-generated small errors were simulated to show the effect on the estimation error of angle *α* from Equation (5) and the distances $p_{B2}$ and $p_{D4}$ from Equation (4). Various constellations of line position and slope (π/2−α) were simulated. Figure 5 presents the results for line position $p_{B3}=\frac{\sqrt{2}}{3}\;\cdot \;b$.

The graph indicates that due to the nonlinearity of arcsin in Equation (5), small errors of 3D feature point extraction result in greater angle errors at slopes within the range of ±20${}^{\circ }$. Consequently, the position errors of $p_{2}^{o}$ and $p_{4}^{o}$ in object coordinates, calculated via Equations (6) and (7), are no more suitable for calibration. By contrast, small errors do not influence the calculation of $p_{3}^{o}$ significantly.

#### 4.3.2. Recovering the Feature Point in the Image

After determining points $p_{i}^{o}$ in object coordinates, the feature points are projected in the camera image. To project an arbitrary point of the calibration front plane ($p_{i,x}^{o}=0$) to the image plane, a homography matrix H is estimated. This homography matrix maps those points on image coordinates as follows:

(8) $$\lambda \left(\begin{array}{c} u_{i}\\ v_{i}\\ 1 \end{array}\right)=H\left(\begin{array}{c} p_{i,y}^{o}\\ p_{i,z}^{o}\\ 1 \end{array}\right)$$

In order to estimate the homography matrix, at least four corresponding points of the planes are needed [18]. This is possible by using the circular rings, since the positions of their centers are known in object coordinates and are found in image coordinates. For this purpose, contours are detected in a binary image according to [19], and elliptical shapes are extracted. As various camera perspectives are expected, it is more suitable to detect ellipses than circles. In addition, using rings makes it easier to filter filled circles that can occur because of inhomogeneous illumination and shading. Moreover, the limitation that the outer and inner contours of a ring have similar ratios of distance between the major and minor axis improves the robustness of the circular marker detection.

Finally, the corresponding 2D points can be written as unit vectors, i.e., feature vectors,

(9) $$f_{i}^{c}=\frac{K^{-1}{\left(\begin{array}{ccc} u_{i} & v_{i} & 1 \end{array}\right)}^{T}}{∥K^{-1}{\left(\begin{array}{ccc} u_{i} & v_{i} & 1 \end{array}\right)}^{T}∥}=\frac{p_{i}^{c}}{∥p_{i}^{c}∥}$$

where the intrinsic camera matrix is represented by K.

## 5. Ego-Motion Estimation

After a successful calibration, it is possible to add 3D information of the laser rangefinder to certain pixels of the camera. The transformation of measured world points ${\tilde{w}}_{i}^{l_{k_{0}}}$ at time $k_{0}$ in laser coordinates to camera coordinates satisfies the following equation:

(10) $${\tilde{w}}_{i}^{c_{k_{0}}}=C_{l}^{c}\left({\tilde{w}}_{i}^{l_{k_{0}}}-t_{l_{k_{0}}c_{k_{0}}}^{l_{k_{0}}}\right)$$

In order to get the corresponding 2D information at time *k*, the world points of Equation (10) are tracked. Since it is not guaranteed that feature points, like SURF [20], lie along the laser-line, feature-based trackers are not suitable for this purpose. Therefore, the pixels with 3D information are tracked by the Kanade-Lucas-Tomasi feature tracker (KLT) [21] and described as feature vectors by:

(11) $${\tilde{f}}_{i}^{c_{k}}=\frac{K^{-1}{\left(\begin{array}{ccc} {\tilde{u}}_{i} & {\tilde{v}}_{i} & 1 \end{array}\right)}^{T}}{∥K^{-1}{\left(\begin{array}{ccc} {\tilde{u}}_{i} & {\tilde{v}}_{i} & 1 \end{array}\right)}^{T}∥}=\frac{w_{i}^{c_{k}}}{∥w_{i}^{c_{k}}∥}$$

whereas K represents the intrinsic matrix.

**Figure 6:**
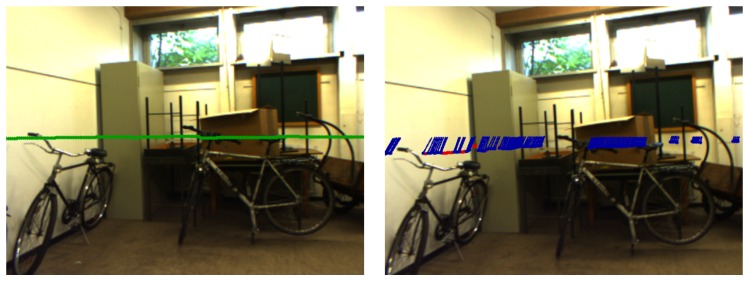
3D information of the laser rangefinder is projected into the image at reference time $k_{0}$ (**left**), and the corresponding pixels are tracked until time *k* (**right**).

Thus, 3D-to-2D correspondences, i.e., ${\tilde{w}}_{i}^{c_{k_{0}}}$ and ${\tilde{f}}_{i}^{c_{k}}$, are established, which provide the input for the P3P algorithm. The world points ${\tilde{w}}_{i}^{c_{k_{0}}}$ provide the relative positioning at time $k_{0}$, whereas the feature vectors ${\tilde{f}}_{i}^{c_{k}}$, also called unit vectors, bearing vectors or directional vectors [15], provide the two 2D information at time *k*. Therefore, the metrical relationship at time $k_{0}$ and the angular relationship at time *k* between the observed control points is given in order to establish the P3P problem [10]. Figure 6 visualizes this approach at time $k_{0}$ and current time *k*.

The left image shows the projection of laser data according to Equation (10). On the other hand, the right image illustrates the tracking by KLT, whereas the P3P-RANSAC inliers are marked blue. Those inliers are used for the Levenberg–Marquardt algorithm [22]. Moreover, the initial estimation for the optimization process is given by the best P3P model. Eventually, the resulting ego-motion, $C_{c_{k}}^{c_{k_{0}}}$ and $t_{c_{k_{0}}c_{k}}^{c_{k_{0}}}$, is integrated in the navigation system, which is described in the next section.

## 6. Integrated Navigation System

**Figure 7:**
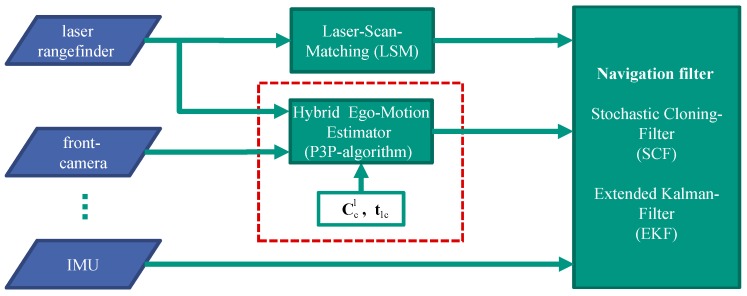
Different sensors and algorithms, including the hybrid ego-motion estimator, aid the navigation filter.

The objective of this section is to integrate the calibrated hybrid ego-motion estimator with the given navigation system; see Figure 7. Typically, the navigation system is an Extended Kalman Filter (EKF). Thereby, the acceleration and angular rate information provided by the IMU is used by the strapdown algorithm at the prediction step. Additionally, the navigation system is updated by further sensor information. However, it is not possible to update the classical EKF with relative positioning and attitude measurements. In order to correctly integrate relative measurements, the navigation system is augmented with the Stochastic Cloning Filter (SCF) approach [23].

The main idea of SCF is to augment the state and its corresponding covariance at time $k_{0}$ as follows:

(12) $$\begin{array}{c} {\overset{ˇ}{x}}_{k_{0}}=\left(\begin{array}{c} x_{k_{0}}\\ x_{k_{0}} \end{array}\right),\;{\overset{ˇ}{P}}_{k_{0}}=\left(\begin{array}{cc} P_{k_{0}} & P_{k_{0}}\\ P_{k_{0}} & P_{k_{0}} \end{array}\right) \end{array}$$

The absolute state is defined as:

(13) $$x_{k}={\left(\begin{array}{ccccc} p^{T} & v^{T} & \Psi^{T} & b_{a}^{T} & b_{\omega }^{T} \end{array}\right)}^{T}$$

whereas the position, velocity, attitude and inertial bias are estimated.

After the filter propagation step, the state and its corresponding covariance can be written as:

(14) $$\begin{array}{c} {\overset{ˇ}{x}}_{k}=\left(\begin{array}{c} x_{k}\\ x_{k_{0}} \end{array}\right)\;\;\mathrm{and}\;\;{\overset{ˇ}{P}}_{k}=\left(\begin{array}{cc} P_{kk} & P_{kk_{0}}\\ P_{k_{0}k} & P_{k_{0}k_{0}} \end{array}\right) \end{array}$$

so that the correlation between state $x_{k_{0}}$ and $x_{k}$ is established. As a consequence, the state can be corrected by relative measurements.

Using frame-by-frame motion estimation, the clone would be renewed permanently. As a consequence, the relative measurements and their errors would be directly accumulated. In order to use the SCF optimally, it is desirable to retain the clone as long as possible. Due to the front-facing camera, feature points can be observed for a long period of time. Therefore, a keyframe-by-frame motion estimation is used. At reference time $k_{0}$, the keyframe is retained, and the state is cloned according to Equation (12). To each subsequent image, the relative pose to the reference frame is derived. This procedure can mitigate the increasing of the uncertainty of the estimated state.

### Covariance Estimation

Since the correction step of Kalman-based navigation filters requires not only measurement, but also information about its uncertainty, this section deals with the covariance estimation. In this case, the covariance of the parameter vector:

(15) $$\theta^{b}={\left(\begin{array}{cccccc} t_{b_{k_{0}}b_{k},x}^{b_{k_{0}}} & t_{b_{k_{0}}b_{k},y}^{b_{k_{0}}} & t_{b_{k_{0}}b_{k},z}^{b_{k_{0}}} & \phi & \theta & \psi \end{array}\right)}^{T}$$

is estimated on the basis of backward propagation described in [24]. Using this parameter vector, i.e., the relative motion estimation obtained by the P3P algorithm, the *n* feature vectors ${\hat{f}}_{i}^{b_{k}}$ (compare Section 5) can be estimated by the following mapping:

(16) $${\hat{f}}_{i}^{b_{k}}:R^{6}\to R^{3},\;\theta^{b}\mapsto \frac{C_{b_{k_{0}}}^{b_{k}}\left({\tilde{w}}_{i}^{b_{k_{0}}}-t_{b_{k_{0}}b_{k}}^{b_{k_{0}}}\right)}{∥C_{b_{k_{0}}}^{b_{k}}\left({\tilde{w}}_{i}^{b_{k_{0}}}-t_{b_{k_{0}}b_{k}}^{b_{k_{0}}}\right)∥}$$

The covariance of the total feature vector ${\hat{f}}^{b_{k}}\;:\;R^{6}\;\to \;R^{3n}$ is approximated by:

(17) $$P_{\hat{f}}=\left(JP_{\theta }J^{T}\right),$$

where the Jacobian J is defined by $J={\left(\begin{array}{ccccc} J_{1}^{T} & \cdots & J_{i}^{T} & \cdots & J_{n}^{T} \end{array}\right)}^{T}$ with $J_{i}=\frac{\partial {\hat{f_{i}}}^{b_{k}}}{\partial \theta^{b}}{|}_{\theta^{b}={\hat{\theta }}^{b}}\in R^{3\times 6}$.

As the covariance of parameter vector $\theta^{b}$ is required, the back propagation:

(18) $$P_{\theta }={\left(J^{T}P_{\hat{f}}^{-1}J\right)}^{-1}\in R^{6\times 6}$$

gives a good approximation of the desired covariance matrix [24]. Assuming that particularly each of the estimation errors of ${\hat{f}}_{i}$ are uncorrelated with each other, the covariance matrix is simplified as $P_{\hat{f}}=\sigma_{\hat{f}}^{2}I$. In order to estimate $\sigma_{\hat{f}}^{2}$, the sample covariance of the reprojection errors $\varepsilon ={\left(\begin{array}{ccccc} \varepsilon_{1} & \cdots & \varepsilon_{i} & \cdots & \varepsilon_{n} \end{array}\right)}^{T}$ with $\varepsilon_{i}=∥{\tilde{f}}_{i}^{b_{k}}\times {\hat{f}}_{i}^{b_{k}}∥$ is calculated.

## 7. Results

The various aspects from calibration, to ego-motion estimation, to covariance estimation, to the final integrated navigation system are studied in the next sections.

### 7.1. Calibration

The proposed calibration method is validated by simulation. Then, the experimental results of calibrating two laser-camera systems confirm the simulation findings.

#### 7.1.1. Simulation

In the first step, our calibration method was evaluated by simulation. As an exact ground truth pose between the laser and camera is given, accuracy information can be gained. Table 1 shows the simulated laser-camera constellation.

**Figure 8:**
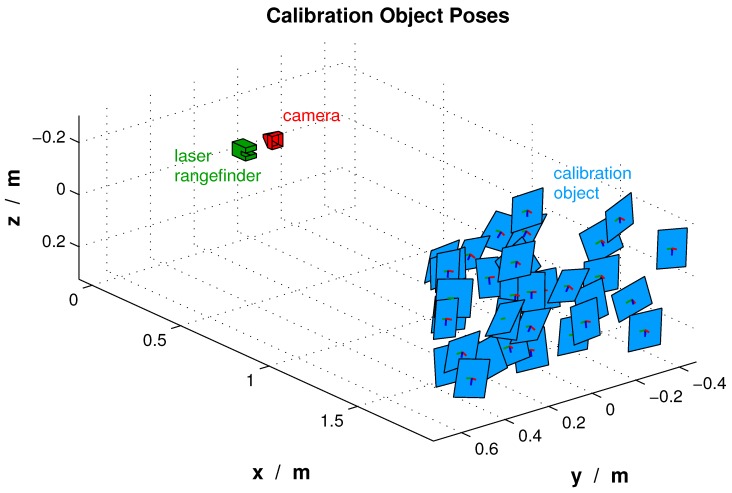
Various calibration poses for the 3D-to-2D correspondences.

The designed 3D model of the calibration object was rendered by the simulation framework. Hence, for each pose between the MAV and the calibration object, simulated sensor data were obtained. A visual representation of the generated poses is illustrated in Figure 8.

**Figure 9:**
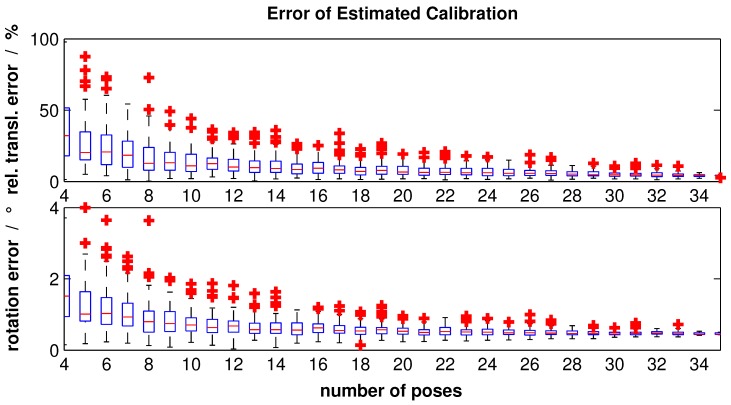
Error analysis of the simulated calibration for each number of poses.

For each number of calibration poses, 100 calibration runs were performed. The error analysis of estimated translation ${\hat{t}}_{lc}^{l}$ and rotation ${\hat{C}}_{c}^{l}$ can be seen as the boxplot in Figure 9. The analysis is done similar to that in [8].

The red horizontal lines represent the median; the borders of the boxes are the first and third quartiles, respectively. Furthermore, the red crosses can be seen as outliers that are outside the 2.7*σ* range. The upper part of Figure 9 shows the relative translation error described as follows:

(19) $$\delta_{t}=\frac{∥\Delta t_{lc}^{l}∥}{∥{\bar{t}}_{lc}^{l}∥}=\frac{∥{\hat{t^{l}}}_{lc}-{\bar{t}}_{lc}^{l}∥}{∥{\bar{t}}_{lc}^{l}∥}$$

The part below outlines the rotation error calculated by the metric:

(20) $$\Phi \left(q_{1},q_{2}\right)=2\;\cdot \;\arccos\left(\left|q_{1}^{T}q_{2}\right|\right)$$

whereas $q_{1}$ and $q_{2}$ are quaternions [25]. This metric compares the estimated quaternion $q_{1}={\hat{q}}_{c}^{l}$ with the reference quaternion $q_{2}={\bar{q}}_{c}^{l}$ and gives values in the range $\left[0,\pi \right)$.

Obviously, the errors and their deviations decrease by increasing the number of poses. Using all poses, a median relative translation error of 2.49% and median rotation error of 0.16${}^{\circ }$ can be achieved with our modified calibration object. In comparison to that, using the same poses with the original calibration object supposed by Hoang et al. [9], relative translation error and rotation error are 4.28% and 0.46${}^{\circ }$, respectively. Likewise, the resulting deviation, namely the box width, for the modified calibration object is smaller than the original one.

In addition, the absolute pose errors between the laser rangefinder and camera presented in Table 2 agree with the previous observation. Considering the rotation angles separately, the pitch angle *θ* is reduced remarkably.

When distances $p_{23}$ and $p_{34}$ are estimated too short, the point $p_{3}^{o}$ calculated via Equations (2) and (3) is estimated too low. This tends to happen very often for the original calibration object. Thus, a bigger pitch angle error for the given sensor constellation is caused. In this context, the distance errors $\Delta p_{23}$ were calculated, and their influence on the distance errors $\Delta p_{B3}$ was observed. Table 3 shows the median of the distance errors. It indicates that the modifications improve the calibration result by reducing $\Delta p_{23}$.

#### 7.1.2. Experimental

**Figure 10:**
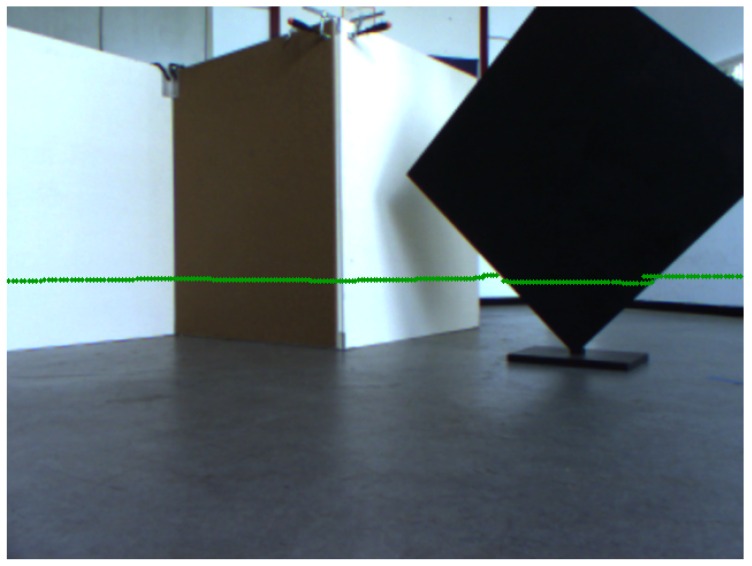
Laser projection into the monocular camera of the MAV.

After obtaining successful simulation results, the laser rangefinder and camera of the MAV (compare Section 2) were calibrated. Since the position and orientation of the sensor coordinate systems cannot be accurately determined, ground truth pose information does not exist. However, the projection of laser scans with the given pose parameters found in Figure 10 shows plausible calibration results. The endpoints of the laser lines are aligned to the edges of the objects.

**Figure 11:**
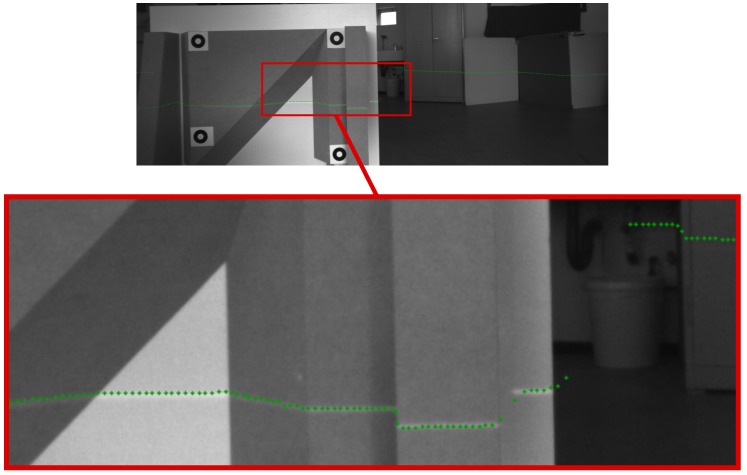
Laser projection into a camera of the Multi-Sensor Pedestrian Navigation System (MSPNS) that detects the laser line.

Beyond that, the calibration method was performed with the Multi-Sensor Pedestrian Navigation System (MSPNS) consisting of a camera and laser rangefinder. This system is applied for pedestrian navigation [26]. In this case, the imager detects also near-infrared (NIR) wavelengths, as described in [27]. Hence, the reflecting laser points are visible in the image. This can be compared to the laser projection transformed by the given calibration result. Thus, Figure 11 confirms the previous observations.

### 7.2. Ego-Motion Estimation

**Figure 12:**
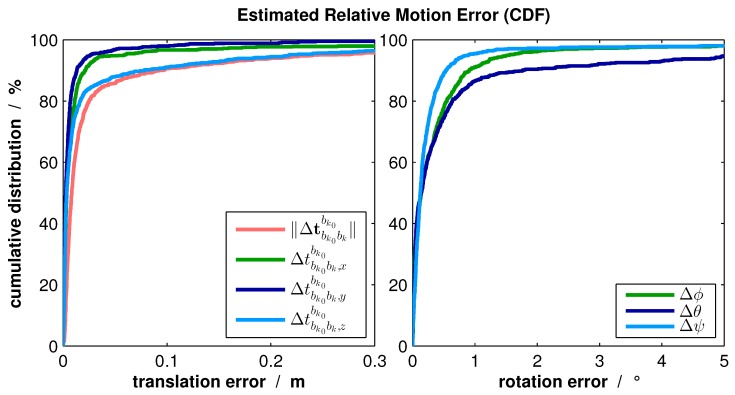
Ego-motion estimation errors occurring in a real indoor flight (CDF).

The ego-motion estimation was separately evaluated with a real indoor-flight along a corridor. The relative poses between the subsequent camera frames were estimated. In order to gain ground truth information, a Kalman filter smoother, based on [28] and presented in [17], was used. This approach provides very accurate navigation solutions by reprocessing the flight in both the forward and backward direction. However, this method is not suitable for online processing due to high computational costs. The resulting estimation errors illustrated as a Cumulative Distribution Function (CDF) can be found in Figure 12.

From this figure, it can be seen that 80% of translation errors $∥\Delta t_{b_{k_{0}}b_{k}}^{b_{k_{0}}}∥$ are below 2 cm. Considering the translation components separately, the horizontal components ${\hat{t}}_{b_{k_{0}}b_{k},x}^{b_{k_{0}}}$ and ${\hat{t}}_{b_{k_{0}}b_{k},y}^{b_{k_{0}}}$ are significantly more accurate. This observation can be explained by the homogenous structure of walls in the vertical direction, which results in greater uncertainty of the KLT tracker. Despite being still accurate in the vertical direction, the total navigation system will be aided additionally by an altimeter and laser measurement directed to the floor.

The right part of Figure 12 demonstrates that the rotation errors are also small. About 80% of those errors are below 0.65${}^{\circ }$. Due to the same reason as described before, the estimation of horizontal component *ψ* is the most accurate.

### 7.3. Integrated Navigation System

This section deals with the evaluation of the proposed covariance estimation. Moreover, the performance of the integrated navigation system is studied and is compared to an existing laser-scan-matching approach.

The following Table 4 shows the sampling rates of the sensors used.

#### 7.3.1. Covariance Estimation

**Figure 13:**
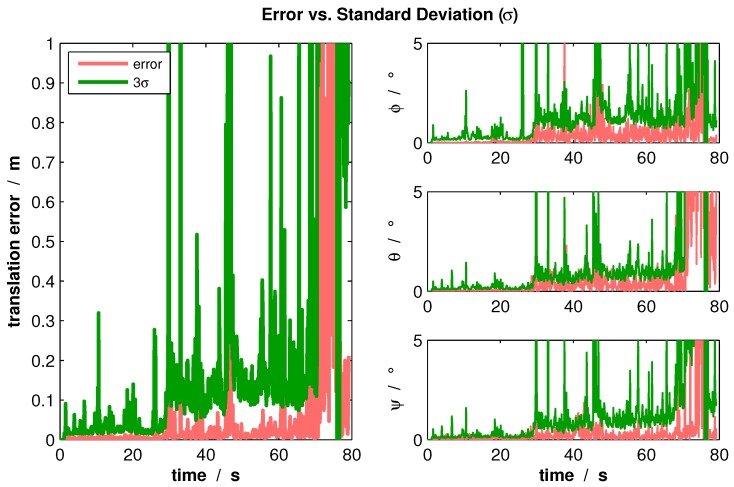
Ego-motion estimation errors occurring in a real indoor flight and the estimated uncertainties.

Two experiments were carried out to analyze the covariance estimation. The first experiment was a real indoor flight along a corridor (compare Section 7.2). Using the Euclidean distance for translation components and considering the rotation components separately, the resulting Figure 13 shows the comparison between the estimation errors and estimated uncertainties.

From this figure, it can be seen that the covariance estimation represents accurately the occurring errors. Furthermore, the graph shows that at the end of the flight, the estimation errors increase rapidly. This happens as the MAV is positioned in front of a wall, where only collinear control points exist. As soon as this degenerated situation occurs, the uncertainty increases accordingly so that the state is corrected by other sensors and methods.

**Figure 14:**
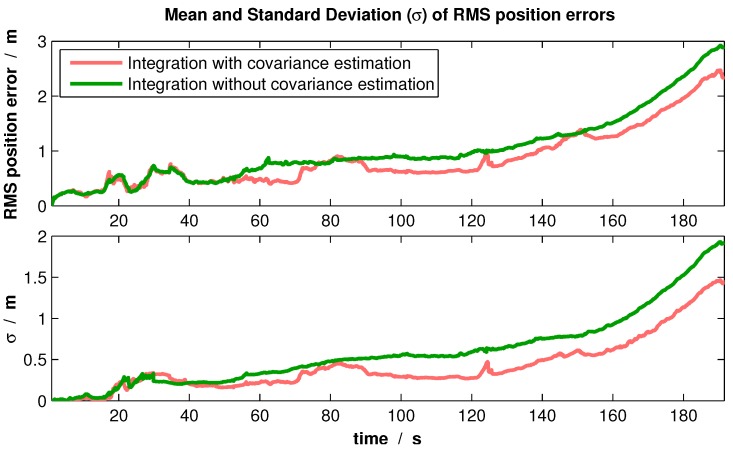
Mean and standard deviation of the RMS position error by reprocessing a real flight multiple times.

The second experiment was a real flight with an outdoor-indoor transition, which comprises different environments. In order to compare the variable covariance estimation with fixed values of uncertainty, this flight was reprocessed in each case 300 times. The root-mean-square errors (RMSE) of the position over time were logged. As a result, the comparison of the mean and standard deviation of those errors is presented in Figure 14.

From the resulting plot, it can be seen that integrating the relative measurements with the introduced covariance estimation improves the position error and, particularly, its variation.

#### 7.3.2. Positioning

In order to evaluate the navigation system, the INS was aided by either the hybrid sensor measurements or by the Laser-Scan-Matching (LSM) approach [4], so that a comparison can be done.

**Figure 15:**
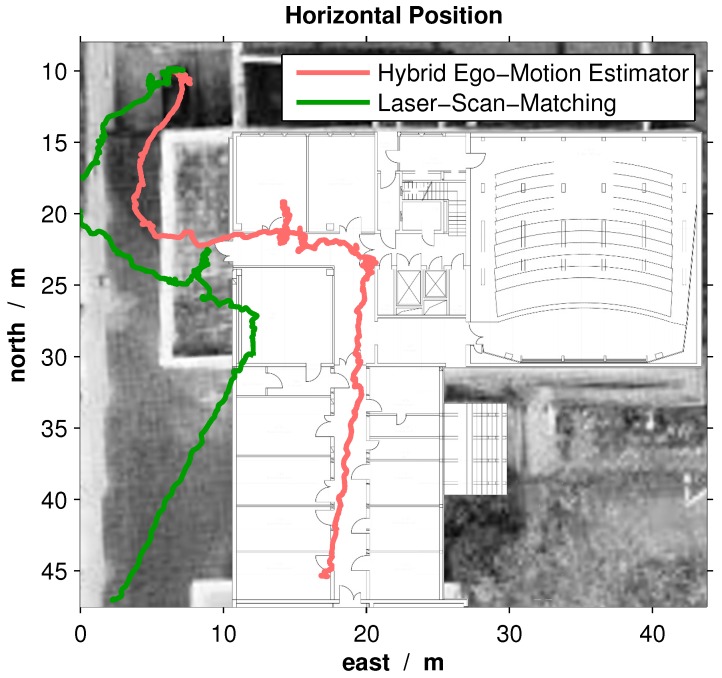
Reprocessed trajectories of the Laser-Scan-Matching (LSM)-aiding and hybrid sensor-aiding system, respectively. The real flight began outside of the building and ended at the end of the corridor. The starting point and the initial orientation of the MAV are measured. Moreover, the building plan with its constraints, e.g., doors and corridor, gives an idea of the ground truth trajectory. Aerial image: © Stadt Karlsruhe.

The experimental flight comprises an outdoor-indoor transition. It starts outdoors, enters the building and flies along the corridor. The start position and initial heading were measured so that absolute position information exists. This results in the trajectories, which are depicted in Figure 15. Starting in the same position and with the same heading, the final position of the MAV using the LSM approach differs significantly from the one using the hybrid sensor-aided navigation system.

The transition of two doors and the corridor itself are good indicators of the estimation errors. Thus, both trajectories were transformed so that these constraints are fulfilled. Since it is known that the LSM approach yields good results in indoor environments, the transformation gives an idea of the performance in outdoor environments. The transformation shifts the starting point and rotates the initial heading so that the indoor constraints are fulfilled. The resulting translation of the starting point and rotation of the initial heading can be found in Table 5.

The resulting trajectories indicate that both integrated navigation systems perform accurately in indoor environments. Thus, the greater heading error of the LSM approach occurs obviously outdoors. This observation shows that the hybrid ego-motion estimator circumvents the limitation of the orthogonal structure of the MAV’s surroundings.

## 8. Conclusions

This article presents a hybrid sensor combining a mono-camera and a 2D laser rangefinder for navigation aiding. This includes many aspects, such as an improved calibration method, ego-motion estimation and the MAV’s integrated navigation system. In this context, attention was focused on being more flexible in terms of environment compared to an existing laser-scan-matching approach. The improved calibration method results in very accurate pose information by both simulation and experiments. After successful calibration of the hybrid sensor system, the six degrees of freedom motion of the MAV have been estimated. Both calibration and ego-motion estimation use 3D-to-2D point correspondences, which are input for the P3P algorithm in the RANSAC scheme. The estimated relative motion of the MAV is integrated by using the stochastic cloning filter technique. In addition, the covariance estimation of the relative measurement is proposed. It shows that the navigation solution becomes more accurate and robust. Comparing the presented hybrid motion estimator with the existing laser-scan-matching approach, it can be concluded that a more flexible solution is found. The hybrid motion estimator can also be used in more unstructured areas, where GNSS is not available or applicable.

In order to optimize the system, it is desirable to study the influence of keyframe selection concerning the duration and other criteria. Beyond ego-motion estimation, the proposed system can also be used for environment perception purposes. Accumulating the laser scans, a metric map of the environment can be generated. In addition, interesting high-level objects from the camera perspective can be integrated so that a semantic map is obtained. The size of those object is estimated by the 3D pixels gained by calibration.

## Figures and Tables

**Table 1:** Simulated pose between the laser and camera.

${\bar{t}}_{lc,x}^{l}$	${\bar{t}}_{lc,y}^{l}$	${\bar{t}}_{lc,z}^{l}$	$\bar{\phi }$	$\bar{\theta }$	$\bar{\psi }$
9.5 cm	0 cm	−3.5 cm	90°	0°	90°

**Table 2:** Absolute pose error between the laser rangefinder and front-facing camera.

Calibration Object	$|\Delta t_{lc,x}^{l}|$	$|\Delta t_{lc,y}^{l}|$	$|\Delta t_{lc,z}^{l}|$	|Δϕ|	|Δθ|	|Δψ|
Hoang et al. [9]	2.424 mm	1.885 mm	3.041 mm	0.070${}^{\circ }$	0.432${}^{\circ }$	0.057${}^{\circ }$
Ours	1.812 mm	0.256 mm	1.739 mm	0.078${}^{\circ }$	0.143${}^{\circ }$	0.022${}^{\circ }$

**Table 3:** Error of distance $p_{23}$ (median) and the resulting error of $p_{B3}$ (median).

Calibration Object	$\Delta p_{23}$	$\Delta p_{B3}$
Hoang et al. [9]	−0.62 cm	−0.86 cm
Ours	0.24 cm	0.35 cm

**Table 4:** Sampling rates of the sensors used.

	IMU	Camera	Laser Rangefinder
Sampling rate	333 Hz	10 Hz	40 Hz

**Table 5:** Rotation and translation of trajectories so that the indoor constraints are fulfilled.

Approach	ΔΨ	$\Delta t_{\mathrm{start},n}^{n}$	$\Delta t_{\mathrm{start},e}^{n}$
Laser-Scan-Matching	27${}^{\circ }$	−1 m	−1 m
Hybrid Ego-Motion Estimator	6${}^{\circ }$	−2.25 m	1 m

## References

[1] Martinelli A. Closed-form solution for attitude and speed determination by fusing monocular vision and inertial sensor measurements. Proceedings of the 2011 IEEE International Conference on Robotics and Automation (ICRA).

[2] Schmid K., Lutz P., Tomić T., Mair E., Hirschmüller H. (2014). Autonomous vision-based micro air vehicle for indoor and outdoor navigation. J. Field Robot..

[3] Scaramuzza D., Fraundorfer F. (2011). Visual odometry [Tutorial]. IEEE Robot. Autom. Mag..

[4] Crocoll P., Seibold J., Popp M., Trommer G.F. Indoor navigation for a micro aerial vehicle aided by laser range finder measurements. Proceedings of the European Navigation Conference.

[5] Ying X., Wang G., Mei X., Yang S., Rong J., Zha H. A direct method for the extrinsic calibration of a camera and a line scan LIDAR. Proceedings of the 2014 IEEE International Conference on Mechatronics and Automation (ICMA).

[6] Zhang Q., Pless R. Extrinsic calibration of a camera and laser range finder (improves camera calibration). Proceedings of the 2004 IEEE/RSJ International Conference on Intelligent Robots and Systems (IROS 2004).

[7] Li G., Liu Y., Dong L., Cai X., Zhou D. An algorithm for extrinsic parameters calibration of a camera and a laser range finder using line features. Proceedings of the IEEE/RSJ International Conference on Intelligent Robots and Systems (IROS 2007).

[8] Vasconcelos F., Barreto J., Nunes U. (2012). A minimal solution for the extrinsic calibration of a camera and a laser-rangefinder. IEEE Trans. Pattern Anal. Mach. Intell..

[9] Hoang V.D., Hernández D.C., Jo K.H. (2014). Simple and efficient method for calibration of a camera and 2D laser rangefinder. Intelligent Information and Database Systems.

[10] Fischler M.A., Bolles R.C. (1981). Random sample consensus: A paradigm for model fitting with applications to image analysis and automated cartography. Commun. ACM.

[11] Muhieddine A., Asmar D., Shammas E. Robot localization using a complementary laser/camera filter. Proceedings of the 2014 IEEE International Conference on Robotics and Biomimetics (ROBIO).

[12] Jutzi B., Weinmann M., Meidow J. (2014). Weighted data fusion for UAV-borne 3D mapping with camera and line laser scanner. Int. J. Image Data Fusion.

[13] Bok Y., Choi D.G., Kweon I.S. (2014). Sensor fusion of cameras and a laser for city-scale 3D reconstruction. Sensors.

[14] Popp M., Atman J., Scholz G., Ruppelt J., Trommer G.F. A reduced camera SLAM approach for indoor and outdoor navigation using laser information for landmark initialization and relative motion information. Proceedings of the 2016 International Technical Meeting of the Institute of Navigation.

[15] Kneip L., Scaramuzza D., Siegwart R. A novel parametrization of the perspective-three-point problem for a direct computation of absolute camera position and orientation. Proceedings of the 2011 IEEE Conference on Computer Vision and Pattern Recognition (CVPR).

[16] Yaghobi M., Jadaliha M., Zolghadr J., Norouzi M. Adaptive line extraction algorithm for SLAM application. Proceedings of the IEEE International Conference on Robotics and Biomimetics (ROBIO 2008).

[17] Crocoll P. (2015). Modellbasierte Quadrokopter-Navigation mit Laserstützung. Ph.D. Thesis.

[18] Ma Y. (2004). An Invitation to 3-D Vision: From Images to Geometric Models.

[19] Suzuki S., Keiichi A. (1985). Topological structural analysis of digitized binary images by border following. Comput. Vis. Graph. Image Process..

[20] Bay H., Tuytelaars T., Van Gool L. (2006). Surf: Speeded up robust features. Computer Vision–ECCV 2006.

[21] Lucas B.D., Kanade T. An iterative image registration technique with an application to stereo vision. Proceedings of the 7th international joint conference on Artificial intelligence.

[22] Marquardt D.W. (1963). An algorithm for least-squares estimation of nonlinear parameters. J. Soc. Ind. Appl. Math..

[23] Roumeliotis S., Burdick J. Stochastic cloning: A generalized framework for processing relative state measurements. Proceedings of the IEEE International Conference on Robotics and Automation (ICRA ’02).

[24] Hartley R., Zisserman A. (2003). Multiple View Geometry in Computer Vision.

[25] Huynh D.Q. (2009). Metrics for 3D rotations: Comparison and analysis. J. Math. Imaging Vis..

[26] Ruppelt J., Kronenwett N., Trommer G.F. A novel finite state machine based step detection technique for pedestrian navigation systems. Proceedings of the 2015 International Conference on Indoor Positioning and Indoor Navigation (IPIN).

[27] Ruppelt J., Trommer G.F. A performance demonstration of stereo visual odometry for outdoor areas and in dark indoor environments. Proceedings of the 22nd Saint Petersburg International Conference on Integrated Navigation Systems.

[28] Rauch H.E., Striebel C., Tung F. (1965). Maximum likelihood estimates of linear dynamic systems. AIAA J..

